# Myelopathic Albumosuria

**Published:** 1905-02-04

**Authors:** 


### MYELOPATHIC ALBUMOSURIA.

MANY rare diseases tend to become common as the means available for their identification are more generally put into practice, and Dr. Paget Moffatt is probably correct in saying that Myelopathic Albumosuria is not so uncommon as it would appear. Most medical men are content to believe that the albuminoid bodies coagulable by boiling are the only ones of the kind with which the practical physician has any concern, and it is well to be reminded that this is not so.

The disease in question is characterised, as its name implies, by the presence of albumose in the urine, in association with marrow changes in certain bones. This albumose can be readily distinguished from serum-albumin—the commonest proteid constituent of pathological urines—by the fact that it is coagulated at a temperature of 60° C, dissolves again to a large extent as the boiling-point is reached, and reappears on cooling, while serum-albumin, once precipitated by heat, undergoes no solution as the boiling-point is approached. Similarly, when treated with nitric acid both are precipitated, but whereas the albumose is partly redissolved on boiling the mixture, the precipitation of the albumin is permanent. Therefore whenever an evanescent cloudiness occurs in the process of boiling an urine, the question of Albumosuria deserves to be brought to mind.

The symptomatology of the disease is obscure, but pain in the back and sides, with progressive feebleness and anæmia, are constant features. Spontaneous fracture of the ribs is frequent, and distortion of the vertebræ not uncommon. The conditions with which it is most likely to be confounded, according to the author, are :—1. Osteomalakia; 2. Lumbago and sciatica; 3. Spondylitis deformans; 4. Caries or malignant disease of the spine; 5. Pernicious anæmia; 6. Nephritis; 7. Chyluria (occasionally, when the albumose is spontaneously precipitated). It is a disease of the second half of life: nothing is known of the ætiology; no treatment has been discovered, and life is rarely prolonged for more than a year after the declaration of symptoms.

The pathological changes are limited to the skeleton, and the long bones usually escape, the brunt of the lesions falling upon the ribs, sternum, and vertebral bodies. In them are found multiple gelatinous new growths, consisting chiefly of lymphoid cells with little or no intercellular substance. The salient distinction between this formation and the commoner varieties of sarcoma, with which it conforms in many particulars, is the simultaneous invasion of several parts of the skeleton, with the absence of any tendency to metastasis in other parts of the body.

